# Nonreciprocal scattering and unidirectional cloaking in nonlinear nanoantennas

**DOI:** 10.1515/nanoph-2024-0212

**Published:** 2024-07-29

**Authors:** Heedong Goh, Alex Krasnok, Andrea Alù

**Affiliations:** Photonics Initiative, Advanced Science Research Center, City University of New York, New York, NY 10031, USA; Department of Electrical and Computer Engineering, Florida International University, Miami, FL 33174, USA; Physics Program, Graduate Center, City University of New York, New York, NY 10016, USA

**Keywords:** nonreciprocal, nonlinear, scattering, nanoantennas, cloaking

## Abstract

Reciprocal scatterers necessarily extinguish the same amount of incoming power when excited from opposite directions. This property implies that it is not possible to realize scatterers that are transparent when excited from one direction but that scatter and absorb light for the opposite excitation, limiting opportunities in the context of asymmetric imaging and nanophotonic circuits. This reciprocity constraint may be overcome with an external bias that breaks time-reversal symmetry, posing however challenges in terms of practical implementations and integration. Here, we explore the use of tailored nonlinearities combined with geometric asymmetries in suitably tailored resonant nanoantennas. We demonstrate that, under suitable design conditions, a nonlinear scatterer can be cloaked for one excitation direction, yet strongly scatters when excited at the same frequency and intensity from the opposite direction. This nonreciprocal scattering phenomenon opens opportunities for nonlinear nanophotonics, asymmetric imaging and visibility, all-optical signal processing and directional sensing.

## 1 Introduction

Controlling wave scattering beyond conventional regimes can expand the functionalities of nanophotonic devices. Common examples of anomalous scattering responses are cloaking [1]–[3], superscattering [4]–[6], beam steering [7], asymmetric or power-dependent responses [8], [9], and nonreciprocal scattering [10]. Non-Hermitian and PT -symmetric systems offer other forms of exotic scattering responses, including exceptional points, embedded eigenstates with large radiative Q-factors, and topologically nontrivial states [11]. In this context, direction-selective transparency and cloaking may be exploited for asymmetric imaging and directional sensing and to realize uni-directional data flows in light-based analog photonic computing platforms [12]. Breaking reciprocity in photonic systems is an outstanding research direction, and various strategies have been developed in recent years, e.g., by using an external bias to break time-reversal symmetry in the constitutive tensors [10,13,14] or by breaking time-invariance through suitable forms of time modulation [15–20]. A nonlinear material response, such as an intensity-dependent permittivity, can also break reciprocity in a bias-free and passive approach [21–31]. As a relevant reference for the following discussion, Ref. [32] discusses the fundamentals of nonlinearity-induced nonreciprocity in the context of two-port systems, and the opportunities and limitations of this approach.

## 2 Nonreciprocity and asymmetric scattering cross-sections

While a finite scatterer in an unbounded medium is not limited to two ports, rather it can couple to a continuum of radiation modes, reciprocity has important implications also for scattering phenomena. This can be realized by considering the forward scattered fields for waves incident from opposite directions [10], [33]. In the absence of material loss and hence of absorption, the total scattering cross-section (SCS) of a general scatterer is proportional to its forward scattering [34]

(1) $$\sigma_{\text{scat}}=\frac{4\pi }{k_{0}}Im\left\{f\left(0\right)\right\}.$$

Here, *f*(0) is the normalized forward scattering amplitude, such that $E_{\text{scat}}/E_{0}\cdot {\hat{e}}_{x}=f\left(0\right){\text{e}}^{\text{i}k_{0}r}/r$ , where *E*_0_ is the amplitude of the incident wave, **E**_scat_ is the scattered electric field, and *r* is an observation point distance in the far-field. While the SCS generally changes for different incident directions, reciprocity requires the same total SCS is obtained when the direction is reversed, as expected because the forward scattering coefficients for opposite directions are related through reciprocity [10].

**Figure 1:**
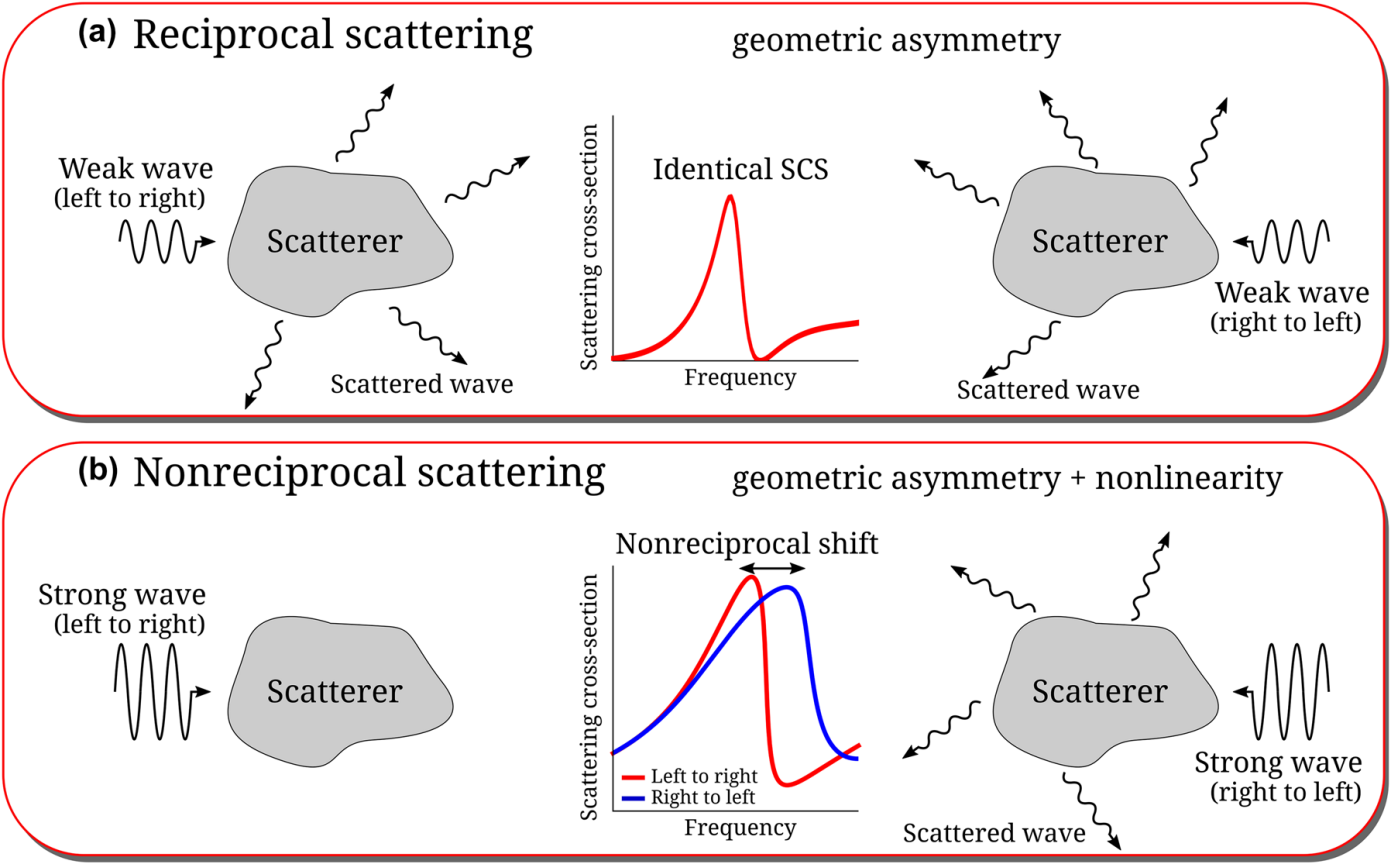
Reciprocal and nonreciprocal scattering. (a) Geometrical asymmetry results in different scattering patterns for different directions of excitations. However, the total scattering cross-section is identical. (b) Nonreciprocal scattering can be achieved by introducing a nonlinear material response in addition to geometric asymmetry.

This symmetry can be expectedly broken by applying a magnetic field in magneto-optical particles [35–40], but magneto-optical phenomena are weak in nanophotonics and often poorly compatible with conventional photonic platforms. Time modulation of the material parameters can also break reciprocity, but it requires a large-intensity optical pump [41]. Using optical nonlinearities combined with geometrical asymmetries may therefore offer unique opportunities for nonreciprocal nanophotonic scattering. Figure 1 shows a schematic of nonreciprocal scattering induced by material nonlinearity. It compares scattering patterns for opposite excitation directions in reciprocal and nonreciprocal scenarios. A geometrically asymmetric object in Figure 1(a) scatters asymmetrically for reversed excitation. However, its total SCS must be the same because of reciprocity [10]. On the other hand, for input intensities sufficiently large to induce optical nonlinearities, an asymmetric frequency shift in the scattering peak can be leveraged to realize nonreciprocal scattering. Figure 1(b) illustrates the extreme scenario of interest here in which cloaking (zero total scattering) is achieved in one direction, while maximum scattering is obtained in the opposite direction. Such a structure therefore consists of a compact scatterer that is transparent upon right-side excitation but, at the same time, it resonantly scatters for left-side excitation at the same frequency and input intensity. This nonreciprocal response may enable novel functionalities in nonlinear photonics, and it may be useful for directional sensors [42] and to enhance the response of nanoscale analog computers [12]. Clouds of such nanoparticles may realize largely asymmetric visibility, blinding an unfriendly observer while enabling visibility from the opposite side.

## 3 Nonreciprocal scattering in a two-particle system

Consider an array of two lossless particles, small enough to be well described by their dipolar polarizabilities *α*_1_ and *α*_2_, which relate the local electric fields to the electric dipole moments **p**_1_ and **p**_2_:

(2) $$\alpha_{1}^{-1}p_{1}=E_{1}^{\text{loc}}\text{ and }\alpha_{2}^{-1}p_{2}=E_{2}^{\text{loc}},$$

where the local electric fields at each particle $E_{1}^{\text{loc}}$ and $E_{2}^{\text{loc}}$ are defined as

(3) $$\begin{array}{ll} E_{1}^{\text{loc}} & =E^{\text{inc}}\left(r_{1}\right)+{\hat{\Gamma }}_{12}p_{2}\;\text{and}\\ E_{2}^{\text{loc}} & =E^{\text{inc}}\left(r_{2}\right)+{\hat{\Gamma }}_{21}p_{1}. \end{array}$$

Here **r**_*i*_ are the positions of the particles and ${\hat{\Gamma }}_{ij}=\left(k_{0}^{2}/\varepsilon_{0}\right)G\left(r_{i},r_{j}\right)$ describe the fields induced by each scatterer on the location of the other scatterer, where **G** is the dyadic Green’s function, *k*_0_ is the wavenumber in a vacuum and *ɛ*_0_ is the vacuum permittivity. We consider a plane wave excitation $E^{\text{inc}}\left(r\right)=E_{0}{\text{e}}^{\text{i}k_{0}r\cos\theta }{\hat{e}}_{x}$ , where *r* is the distance from the origin, *θ* is the polar angle, and *E*_0_ is the amplitude of the incident electric field.

Each particle is designed to exhibit a nonlinear Fano resonance, where we rely on Mie theory to tailor the desired resonant behavior [34]. The polarizabilities of each particle are defined as

(4) $$\alpha_{i}^{-1}=-i\frac{k_{0}^{3}}{6\pi \varepsilon_{0}}\frac{1}{c_{i,1}^{\text{TM}}},$$

where the Mie coefficient $c_{i,1}^{\text{TM}}$ depends on the input intensity through the nonlinear permittivity $\varepsilon_{i}=\varepsilon_{i}^{\text{lin}}+\chi_{i}^{\left(3\right)}{\left|E_{i}^{\text{loc}}\right|}^{2}$ , $\chi_{i}^{\left(3\right)}$ is the third-order susceptibility and $\varepsilon_{i}^{\text{lin}}$ is the linear permittivity. By considering silicon spheres with $\varepsilon_{i}^{\text{lin}}=3.967\varepsilon_{0}$ and $\chi_{i}^{\left(3\right)}=2.8\times 10^{-18}$ m^2^/V^2^ [29], with radii *a*_1_ = 200 nm and *a*_2_ = 230 nm, and a distance between the two particles |*r*_1_ − *r*_2_| = 975 nm, we find a sufficiently asymmetric Fano resonant scattering from the two particles to demonstrate nonreciprocal cloaking for a plane wave excitation with electric field *E*_0_ = 1.8 GV/m (or 430 GW/cm^2^). The selected separation distance between the two particles exceeds the radius of each particle, fulfilling the requirements of the dipole approximation [43]. A detailed discussion of our analytical model, together with its solution method and an inverse design approach, are provided in the Supplementary Materials.

**Figure 2:**
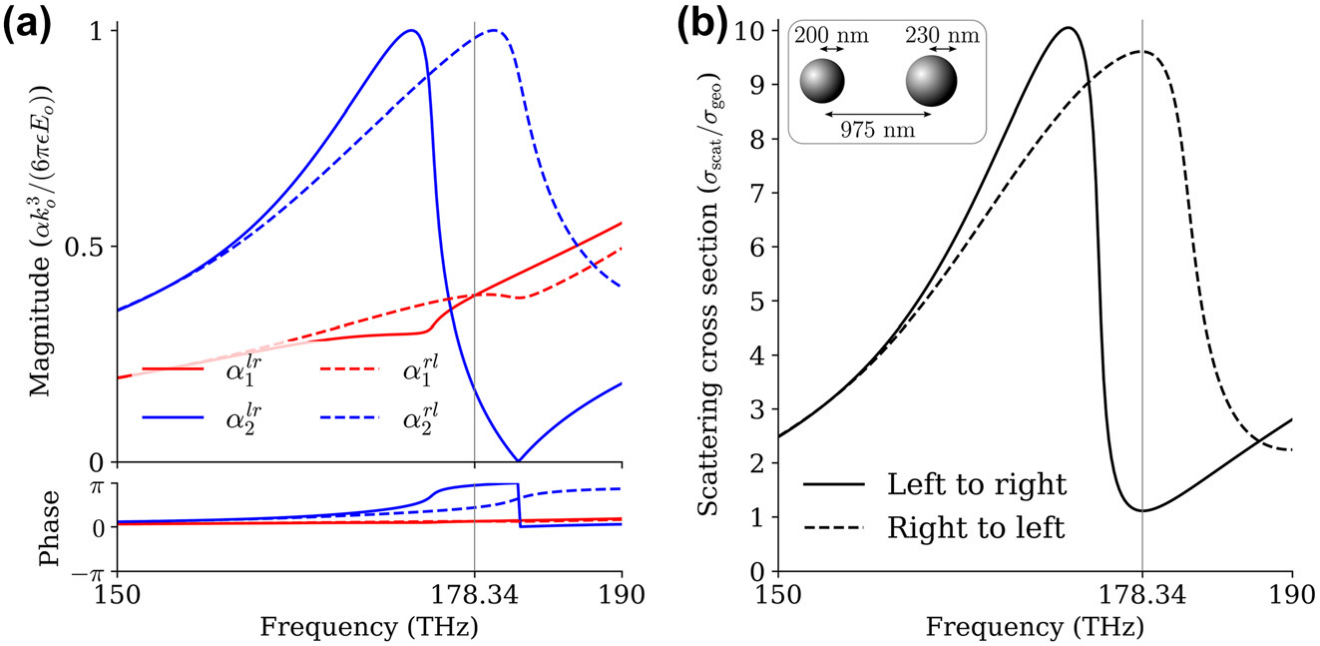
Polarizabilities and scattering cross-sections. (a) Polarizabilities of particle 1 and particle 2 indicated by red and blue lines, respectively, where the solid lines refer to left-to-right (positive *z*-direction) excitation, and dashed lines refer to right-to-left (negative *z*-direction) excitations. (b) Total scattering cross-section (SCS). Large nonreciprocity is observed at the frequency of 178.34 THz: minimum scattering for left-to-right excitation and maximum scattering for right-to-left excitation. The total scattering cross-section is normalized by the geometric cross-section of the largest particle.

Figure 2 shows the calculated polarizabilities (a) and total scattering cross-sections (b) of the two-particle system for opposite excitations as we scan the frequency. We observe the largest SCS contrast at *f* = 178.34 THz, for which we achieve minimum scattering when the incident wave propagates in the positive *z*-direction (left-to-right case), while we find a resonant peak in scattering when the incident wave propagates in the negative *z*-direction (right-to-left case). The polarizability of particle 1 shows a small change for opposite excitation directions because its resonant frequency is above the frequency of interest; conversely, particle 2 shows a significant shift in its resonant frequency near the design frequency. Minimum scattering is achieved when the phases of the induced dipoles are opposite, canceling their dipolar radiation in the far-field. This gives rise to a cloaking response [1]. In the reverse excitation scenario, the dipole moments are instead close to in-phase, leading to a scattering peak. By considering more degrees of freedom in the scattering system, e.g., using different shapes or a larger number of particles, the scattering contrast may be further increased [44,45].

**Figure 3:**
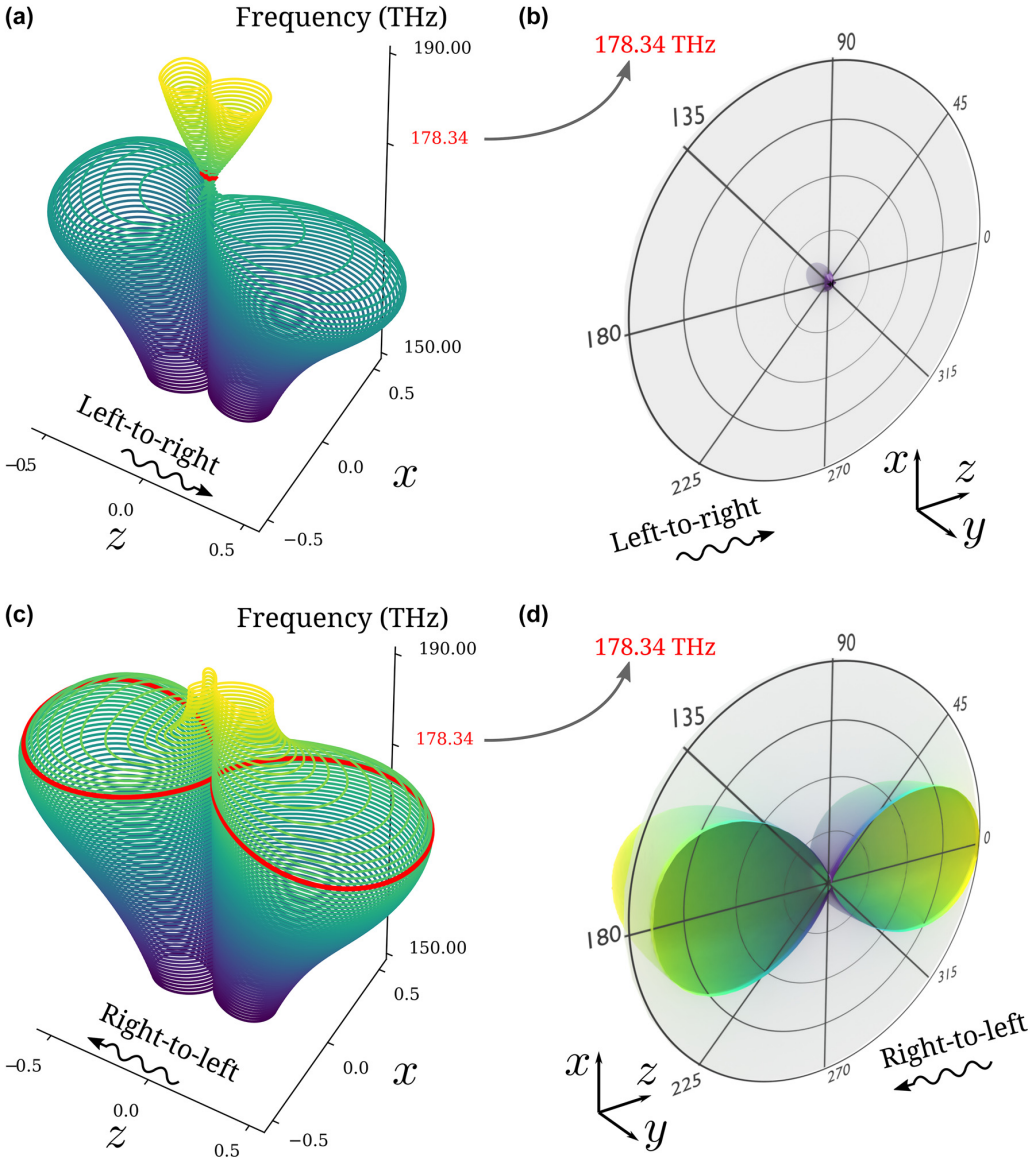
Differential scattering cross-sections. (a) Differential SCS in polar angle (*x*–*z* plane) when the incident planewave propagates from left to right (positive *z*-direction). Minimum scattering is observed at *f* = 178.34 THz (highlighted in red). (b) 3D plot of the differential SCS at *f* = 178.34 THz. The symmetric half is clipped to highlight the scattering pattern in polar angles. (c) Differential SCS in polar angle when the incident wave propagates from right to left (negative *z*-direction). Maximum scattering is observed at *f* = 178.34 THz (highlighted in red). (d) 3D plot of the differential SCS at *f* = 178.34 THz.

Figure 3(a) and (c) show differential SCS patterns in polar angle versus frequency for opposite incident directions. Accordingly, Figure 3(b) and (d) show the three-dimensional differential SCSs clipped in the *x*–*z* plane, at the design frequency. Overall, we observe a dominant dipolar response in the direction of the forward and backward scattering regions. Figure 3(b) demonstrates negligible scattering in the forward and backward regions in contrast to Figure 3(d), and a much smaller overall scattering. On the other hand, differential SCSs in the perpendicular directions are nearly the same for both incidence cases, since they are associated with higher-order harmonics not considered in our design. The corresponding total and scattered electric fields are provided in the Supplementary Materials, demonstrating good unidirectional cloaking performance and dramatic scattering contrast.

**Figure 4:**
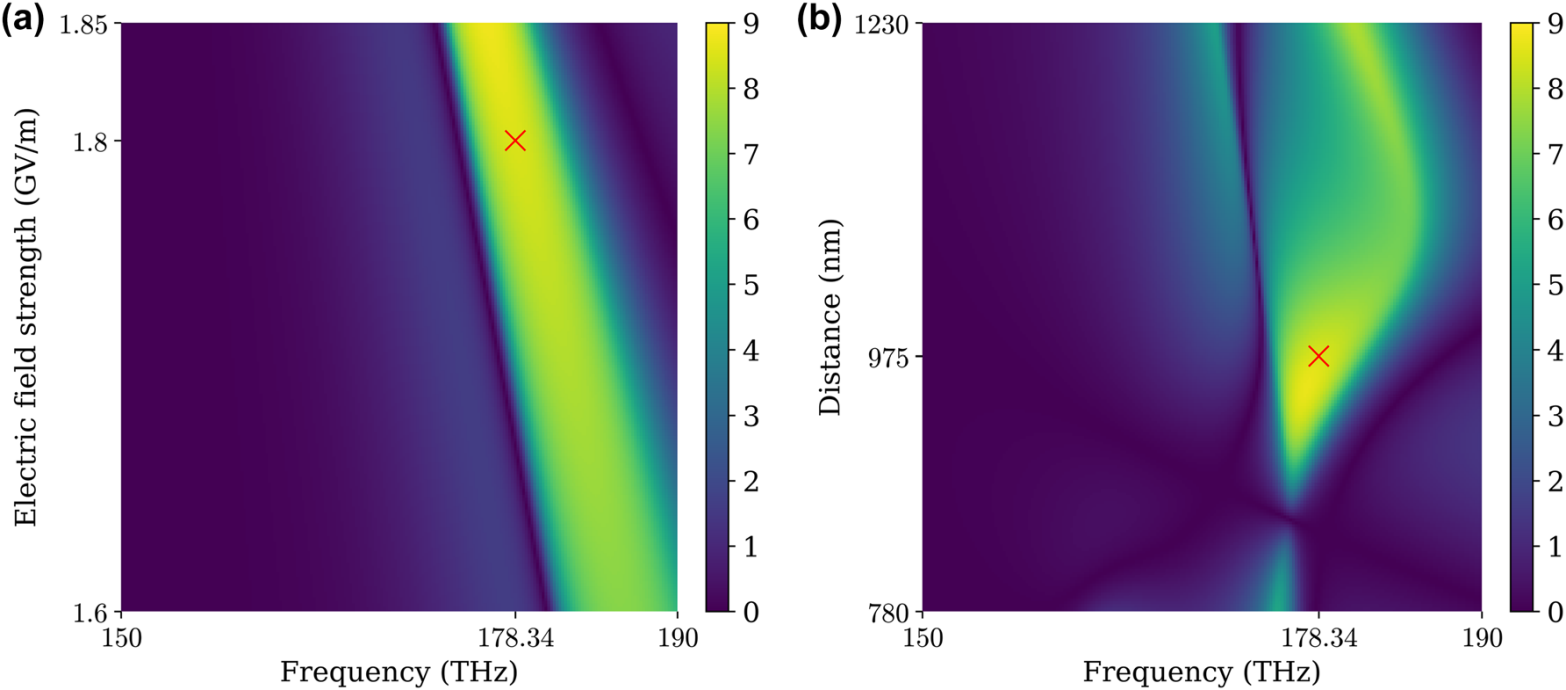
Scattering cross-section contrast, $\left|\sigma_{\text{scat}}^{lr}-\sigma_{\text{scat}}^{rl}\right|/\sigma_{\text{geo}}$ versus (a) incident field strength and frequency and (b) particle distance and frequency. The parameter choices for Figures 2 and 3 are marked by red “×”.

Finally, we performed a parametric study to explore the dependence of the SCS contrast on the electric field strength and frequency [Figure 4(a)] and on the particle distance and frequency [Figure 4(b)]. Figure 4(a) demonstrates that more considerable contrast $\left|\sigma_{\text{scat}}^{lr}-\sigma_{\text{scat}}^{rl}\right|/\sigma_{\text{geo}}$ is achieved for a stronger incident field, while the frequency of the largest contrast monotonically shifts as the incident field strength increases. The SCS contrast is sensitive to the distance between the particles [Figure 4(b)]. Once the particle distance is determined, the design frequency and strength can be determined based on Figure 4(a).

## 4 Conclusions

In this paper we have shown that a geometrically asymmetric subwavelength nonlinear scatterer can support largely nonreciprocal scattering, close to the maximum contrast achievable for a dipolar particle, providing a passive and bias-free approach for nonreciprocal scattering and unidirectional cloaking. Because the approach relies on nonlinearity, the principle of linear superposition cannot be applied, implying that, in the presence of waves from opposite directions, the system may not be able to ‘isolate’ one side. In other words, in the presence of a strong input wave from the ‘uncloaked’ direction, a signal coming from the opposite side may be able to drastically modify the scattering response. The designed response may be of interest for nanophotonic applications, e.g., in the realization of optically controlled transmission switching [32]. A cloud of such particles, properly aligned, may enable efficient asymmetric visibility beyond the limits expected for reciprocal system, e.g., the trade-off between visibility and asymmetry in conventional one-way mirrors. We demonstrated this exotic scattering response using a simple two-particle array of nonlinear Fano dipolar scatterers, and more complex geometries may be optimized using an inverse design approach to achieve more extreme responses, e.g., switchable superscattering and transparency or to enable greater flexibility in the response, of interest to broaden the operator space for wave-based analog computers when combined with the precise control over the scatterer nonlocality [12]. The proposed nonreciprocal scattering features can be used as a building block to design ultrafast switchable nonreciprocal mirrors, metalens, absorbers, metagratings, and photonic topological insulators.

## Supplementary Material

Supplementary Material Details
