# Supplementary material for: Nonreciprocal scattering and unidirectional cloaking in nonlinear nanoantennas
Source: Nanophotonics. 2024 Jul 29;13(18):3347–53. doi: 10.1515/nanoph-2024-0212 (PMC11501511; doi:10.1515/nanoph-2024-0212)
Supplement: Supplementary file 1 — Supplementary Material Details [file j_nanoph-2024-0212_suppl_001.pdf]

Supplementary Materials:

Nonreciprocal Scattering and Unidirectional Cloaking in Nonlinear Nanoantennas

Heedong Goh[1], Alexandr Krasnok[2], and Andrea Alù[1,3,*]

[1]Photonics Initiative, Advanced Science Research Center, City University of New York, New York, NY 10031, USA

[2]Department of Electrical and Computer Engineering, Florida International University, Miami, FL 33174, USA

[3]Physics Program, Graduate Center, City University of New York, New York, NY 10016, USA

*To whom correspondence should be addressed: aalu@gc.cuny.edu

This supplementary material describes the solution method of a nonlinear two-particle system and an inverse design method for the positions of particles and provides additional figures supporting the examples in the main text.

Quasi-Newton method

Here, we introduce a quasi-Newton method to find the dipole moments for given positions and material properties of particles. Once the dipole moments are computed, the computations of scattered field and scattering cross-section are straightforward. We rewrite the Eq. (2) of the main text as

$$\mathbf{0} = \mathbf{f}\left(\mathbf{p}_1, \mathbf{p}_2\right) = \begin{pmatrix} \alpha_1^{-1}\left(\mathbf{p}_2\right)\mathbf{p}_1 - \mathbf{E}_1^{\text{loc}}\left(\mathbf{p}_2\right) \\ \alpha_2^{-1}\left(\mathbf{p}_1\right)\mathbf{p}_2 - \mathbf{E}_2^{\text{loc}}\left(\mathbf{p}_1\right) \end{pmatrix}. \tag{S1}$$

We take the Taylor expansion of the above equation about a trial solution $(\mathbf{p}_{1c}, \mathbf{p}_{2c})$:

$$\begin{aligned} \mathbf{0} &= \mathbf{f}\left(\mathbf{p}_1, \mathbf{p}_2\right) \\ &\approx \mathbf{f}\left(\mathbf{p}_1, \mathbf{p}_2\right)\Big|_{\substack{\mathbf{p}_1=\mathbf{p}_{1c} \\ \mathbf{p}_2=\mathbf{p}_{2c}}} + \left[\frac{\partial}{\partial \mathbf{p}_1}\mathbf{f}\left(\mathbf{p}_1, \mathbf{p}_2\right)\Big|_{\substack{\mathbf{p}_1=\mathbf{p}_{1c} \\ \mathbf{p}_2=\mathbf{p}_{2c}}} \quad \frac{\partial}{\partial \mathbf{p}_2}\mathbf{f}\left(\mathbf{p}_1, \mathbf{p}_2\right)\Big|_{\substack{\mathbf{p}_1=\mathbf{p}_{1c} \\ \mathbf{p}_2=\mathbf{p}_{2c}}} \right] \begin{pmatrix} \mathbf{p}_1 - \mathbf{p}_{1c} \\ \mathbf{p}_2 - \mathbf{p}_{2c} \end{pmatrix} \\ &= \mathbf{r} + \mathbf{K}\Delta\mathbf{x}, \end{aligned} \tag{S2}$$

where

$$\begin{aligned} \frac{\partial}{\partial \mathbf{p}_1}\mathbf{f}\left(\mathbf{p}_1, \mathbf{p}_2\right)\Big|_{\substack{\mathbf{p}_1=\mathbf{p}_{1c} \\ \mathbf{p}_2=\mathbf{p}_{2c}}} &= \begin{bmatrix} \alpha_1^{-1}\Big|_{\mathbf{p}_2=\mathbf{p}_{2c}} \mathbf{I} \\ \dfrac{\partial \alpha_2^{-1}}{\partial \mathbf{p}_1}\Big|_{\mathbf{p}_1=\mathbf{p}_{1c}} \mathbf{p}_{2c} - \mathbf{\Gamma}_{21} \end{bmatrix} \quad \text{and} \\ \frac{\partial}{\partial \mathbf{p}_2}\mathbf{f}\left(\mathbf{p}_1, \mathbf{p}_2\right)\Big|_{\substack{\mathbf{p}_1=\mathbf{p}_{1c} \\ \mathbf{p}_2=\mathbf{p}_{2c}}} &= \begin{bmatrix} \dfrac{\partial \alpha_1^{-1}}{\partial \mathbf{p}_2}\Big|_{\mathbf{p}_2=\mathbf{p}_{2c}} \mathbf{p}_{1c} - \mathbf{\Gamma}_{12} \\ \alpha_2^{-1}\Big|_{\mathbf{p}_1=\mathbf{p}_{1c}} \mathbf{I} \end{bmatrix}. \end{aligned} \tag{S3}$$

Let $\mathbf{x}_c = \left(\mathbf{p}_{1c} \ \ \mathbf{p}_{2c}\right)^{\mathrm{T}}$ and $\mathbf{K}\left(\mathbf{x}_c\right)\Delta\mathbf{x} = -\mathbf{r}\left(\mathbf{x}_c\right)$; then, we update the trial solution as

$$\mathbf{x}_{\text{new}} = \mathbf{x}_c + \Delta\mathbf{x}. \tag{S4}$$

In the above, we inexactly evaluate the gradient by setting $\partial\alpha_i^{-1} / \partial\mathbf{p}_j = \mathbf{0}$, and iterate until the solution is converged.

Design method

Parametric studies may be intractable and require a numerical approach for designing a system with particles more than two. Here, we introduce an inverse problem for designing positions of particles to maximize the scattering contrast. We define a minimization problem:

Given material properties and radii of particles and frequency, find x such that

$$\min\left\{ M\left[x\right] = \frac{1}{2}\frac{\sigma_{\text{geo}}^{2}}{\left\|\sigma_{\text{scat}}^{lr} - \sigma_{\text{scat}}^{rl}\right\|^{2}} : \sigma_{\text{scat}} = \frac{4\pi}{k_0}\operatorname{Im}\left\{f(0)\right\}\right\}. \tag{S5}$$

In the above, the forward scattering $f(0)$ is a function of positions $x = [x_1, y_1, x_2, y_2, \ldots, x_N, y_N]$, where x_i, y_i, $i = 1, 2, \ldots, N$ are the coordinates of each i-th particle and N is the number of particles. Any gradient-based algorithm can be incorporated to solve the above minimization problem when the gradient at a trial solution $x^{(n)}$ is obtained either by automatic differentiation or finite difference method, i.e.,

$$\left.\frac{\partial M\left[x\right]}{\partial x_i}\right|_{x=x^{(n)}} \approx \frac{M\left[x^{(n)} + \varepsilon v_i\right] - M\left[x^{(n)}\right]}{\varepsilon}. \tag{S6}$$

Here, ε is a small number and $v_i = \left[0, \ldots, 0, 1, 0, \ldots, 0\right]$ is a vector of zeros except at i-th entry.

Other supplementary figures

Figure S1 shows scattered and total electric fields for left-to-right and right-to-left excitation cases in x-z plane. The calculations of scattered fields are based on equation 3 of the main text with

the matrix Γ_{ij} generalized for arbitrary positions. We used the same parameter choice used in the main text, i.e., $E_0 = 1.8$ GV/m, $\varepsilon_i^{\text{lin}} = 3.97\varepsilon_0$, $\chi_i^{(3)} = 2.8\times10^{-18}$ m^2/V^2, $a_1 = 200$ nm, $a_2 = 230$ nm, and $|r_1 - r_2| = 975$ nm. We consider two directions of the incident plane waves: 1) left-to-right case (positive z-direction) and 2) right-to-left case (negative z-direction). We observe a negligible scattering of the two-particle system when the incident wave propagates from left to right, while the right-to-left case shows significant scattering. On the other hand, we observe a similar amount of scattering for both cases in the direction perpendicular to the incident wave (Figure S2.) as expected by the differential SCS plot (Figure 3 of the main text).

Figure S3 shows how the total SCS changes as we change the angle of the incident field. The highest scattering contrast appears when the incident directions are in $\pm z$-directions with some angular tolerance.

Figure 4S. repeats the parametric studies of the Figure 4 in the main text with added losses: $\text{Im}\{n\} = 0.05$ for (a) and (b) and $\text{Im}\{n\} = 0.1$ for (c) and (d), where n is the refractive index. The "landscape"s of the contrast remain similar to the cases without losses; however, the strengths of the contrast are decreased with losses.

Figures

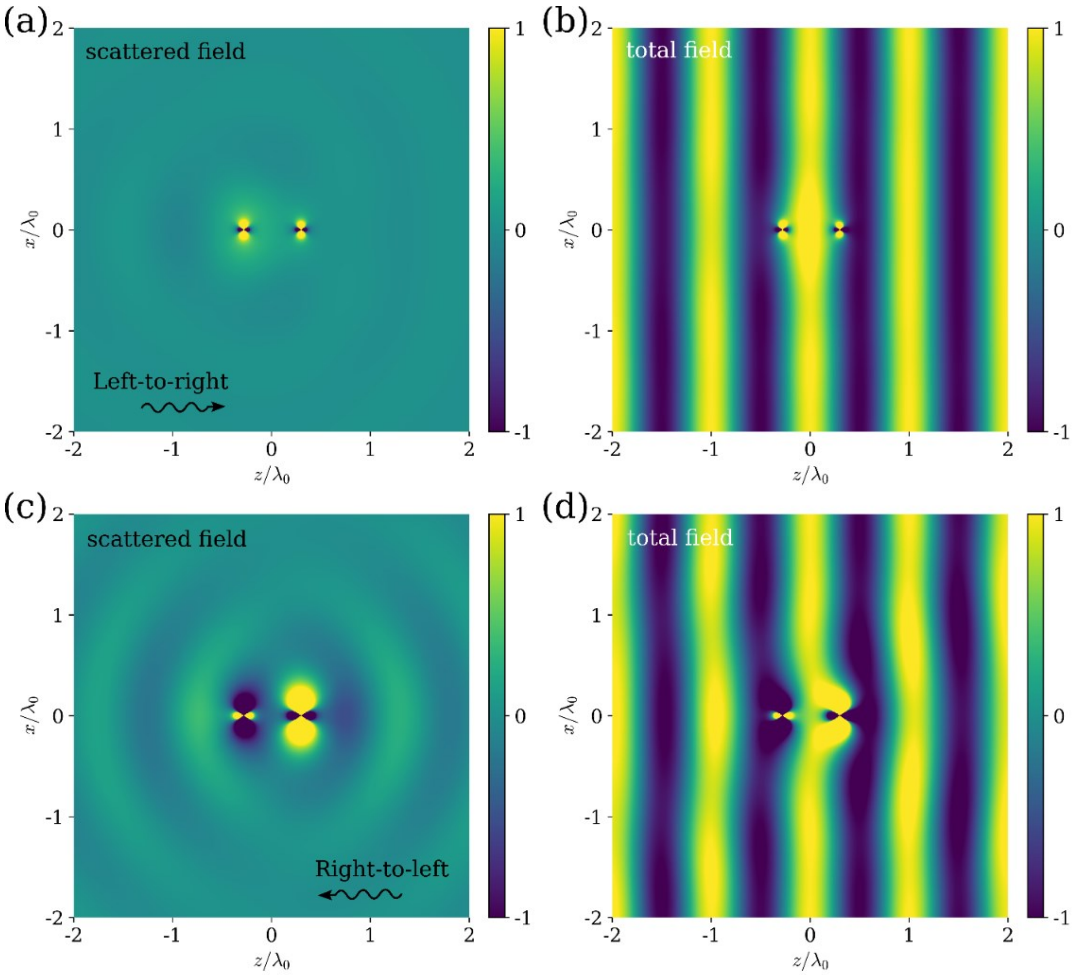

Figure S1. Scattered and total electric fields in x - z plane. (a), (b) scattered and total electric fields for the left-to-right case. (c), (d) scattered and total electric fields for the right-to-left case.

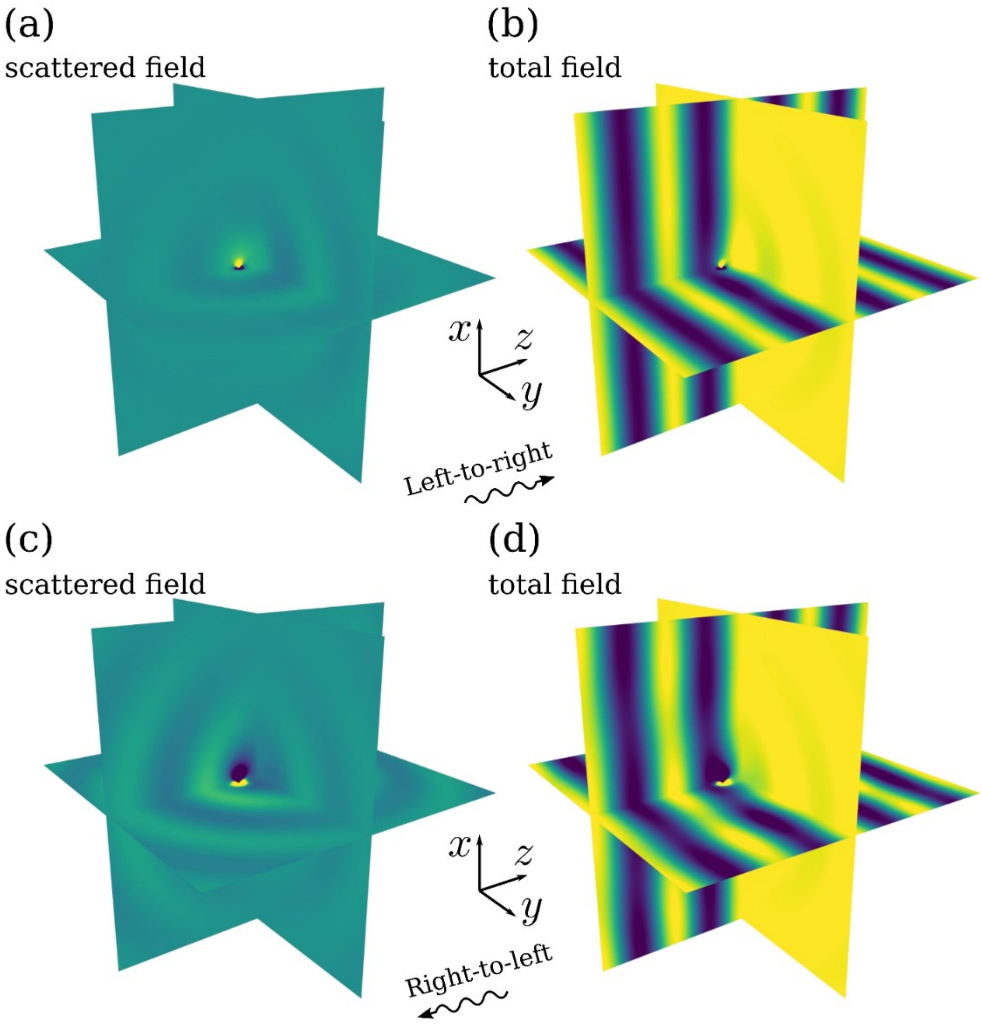

Figure S2. Scattered and total electric fields in x - y , y - z , and z - x planes. (a), (b) scattered and total electric fields for the left-to-right case. (c), (d) scattered and total electric fields for the right-to-left case.

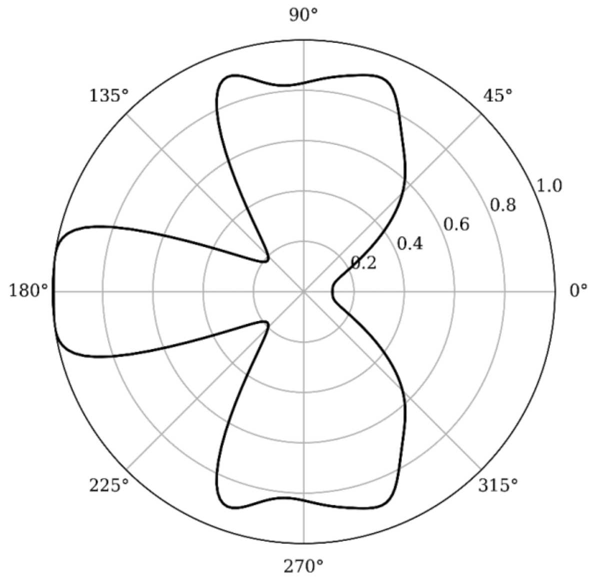

Figure S3. Total scattering cross-section (SCS) versus incident angle. The left-to-right case corresponds to 0°, while the right-to-left case corresponds to 180°. We observe some angular tolerance for maximum and minimum scattering.

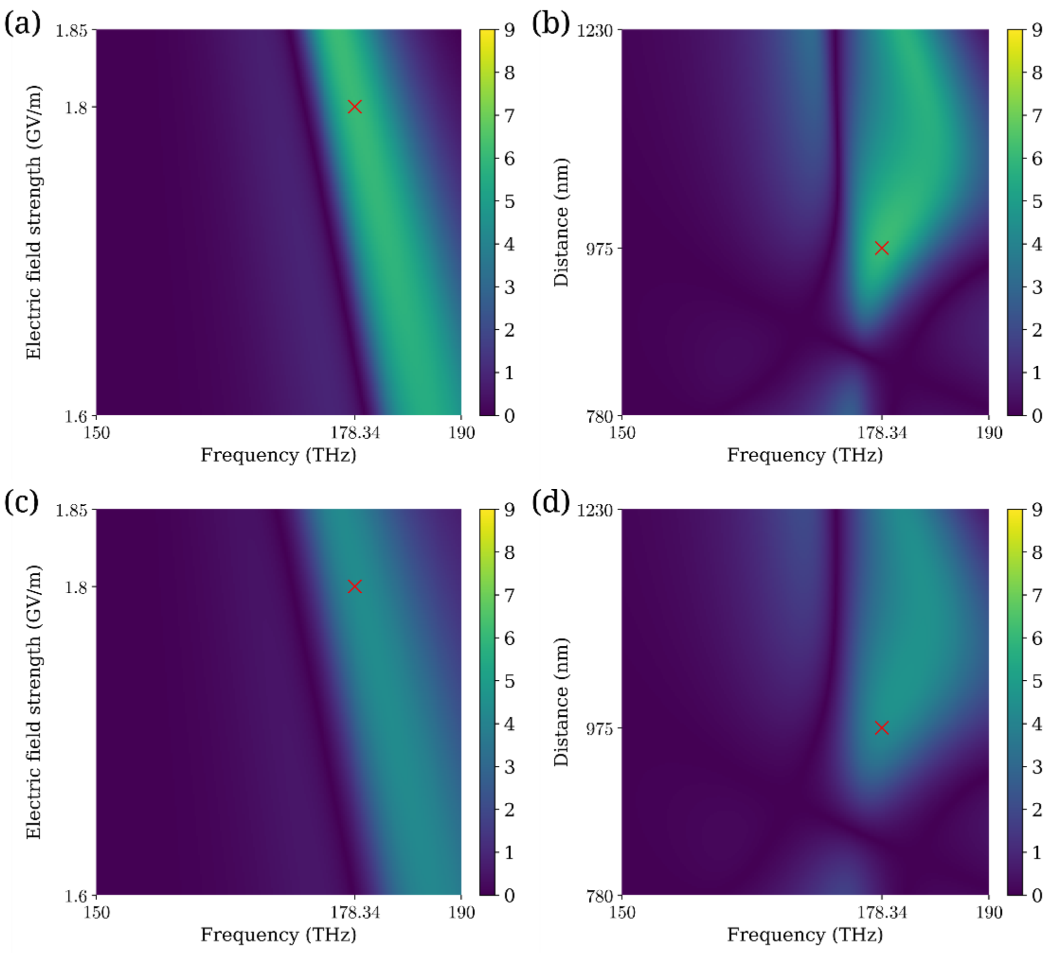

Figure S4. Scattering cross-section contrast, $\left|\sigma_{\text{scat}}^{lr} - \sigma_{\text{scat}}^{rl}\right| / \sigma_{\text{geo}}$ versus (a),(c) incident field strength and frequency and (b),(d) particle distance and frequency. Small losses are applied by complex-valued refractive indices. $\text{Im}\{n\} = 0.05$ for (a),(b) and $\text{Im}\{n\} = 0.1$ for (c),(d) The parameter choices for Figures 2 and 3 of the main text are marked by red "×".
